# Genetic Composition Is Unrelated to Song Content in a Wild Passerine

**DOI:** 10.1111/mec.70491

**Published:** 2026-08-05

**Authors:** Mónika Jablonszky, David Canal, Gergely Hegyi, Gergely Nagy, Balázs Rosivall, János Török, Sándor Zsebők, László Zsolt Garamszegi

**Affiliations:** ^1^ Institute of Ecology and Botany HUN‐REN Centre for Ecological Research Vácrátót Hungary; ^2^ Behavioural Ecology Group, Department of Systematic Zoology and Ecology ELTE Eötvös Loránd University Budapest Hungary

**Keywords:** bioacoustics, bird, genomics, mantel test, quantitative genetics, single nucleotide polymorphism

## Abstract

Birdsong is a key sexual signal that varies substantially among individuals and populations. Understanding whether song similarity reflects genetic similarity is central for evaluating its role in sexual selection, population divergence and evolutionary dynamics, although partial vocal learning can obscure this relationship in many species. Assessing the relationship between song and genetic variation requires accounting for both spatial and temporal heterogeneity in both traits, so despite its importance, the genetic architecture underlying song traits, and in particular song content, remains poorly understood. Here, drawing on 15 years of song recordings and extensive SNP (Single Nucleotide Polymorphism) data, we investigate whether song similarity among males reflects their genome‐wide genetic similarity in the collared flycatcher ( *Ficedula albicollis* ), a migratory passerine with complex and variable songs. After accounting for geographic and temporal separation, we found no strong association between male song similarity and genome‐wide genetic similarity. Moreover, males belonging to the same genetic cluster did not seem to produce more similar songs than those from different clusters. Additionally, genetic distance was unrelated to either geographic or temporal distance, and geographic proximity did not predict song similarity. Instead, song content similarity decreased with increasing temporal separation between recordings, consistent with cultural evolution. Together, these findings indicate that biologically relevant song features are primarily shaped by cultural processes rather than genetic architecture, enabling rapid, flexible responses to environmental changes.

## 1 Introduction

Vocal signals are important and efficient means of communication, as they are diverse enough to convey a wide range of information and can be transmitted over long distances (Rivera‐Gutierrez et al. 2010; Titze and Riede 2010; Aubin and Mathevon 2020). Among vocal signals, bird song is the best studied model due to its central role in intra‐ and intersexual selection (Gil and Gahr 2002; Catchpole and Slater 2008). Although bird song has a genetic basis (Forstmeier et al. 2009; Lewis et al. 2021; Jablonszky, Canal, Hegyi, Herényi, et al. 2022), it is partly learned, to a degree that varies among species (Beecher and Brenowitz 2005; Mets and Brainard 2018). This dual nature introduces uncertainty about the extent to which songs function as reliable signals of genetic quality or compatibility for potential mates (Payne and Westneat 1988; Tregenza and Wedell 2000; Garamszegi et al. 2018) and contribute to sexual selection and population divergence (Lachlan and Servedio 2004; Ribot et al. 2012; Verzijden et al. 2012). A strong link between genetics and song can accelerate divergence, while weak association can hinder evolutionary change in song (Ribot et al. 2012; Verzijden et al. 2012; Ore et al. 2023), and although song learning can decouple song and genetic divergence between populations (Ellers and Slabbekoorn 2003), it may also facilitate speciation if initial song differences emerged between populations (Slabbekoorn and Smith 2002; Lachlan and Servedio 2004; Nicholls and Goldizen 2006). Consequently, the relationship between genetic and song variation is complex and can have major evolutionary implications (Fisher 1930; Ellegren and Sheldon 2008).

The heritability of song has often been examined for spectro‐temporal traits such as song length or frequency (Forstmeier et al. 2009; Labra and Lampe 2018; Jablonszky, Canal, Hegyi, Herényi, et al. 2022), yet these traits typically show low heritability and sometimes unclear biological relevance (Garamszegi and Møller 2004; Hegyi et al. 2010). By contrast, song content—the syllable composition of the songs—have been scarcely studied despite its key role in the context of cultural evolution, syllables and set syllable sequences forming its basis (Lynch and Baker 1993; Podos et al. 2004; Garrido Coria et al. 2025) and sexual selection, as song content can reflect local knowledge, familiarity and can facilitate communication (Wilson et al. 2000; Garamszegi et al. 2012; Nelson and Poesel 2013). Thus, the link between song content and genetic structure can reflect genetic constraints on vocal learning. Unlike continuous or binary traits, song content cannot be analysed with standard quantitative genetic models (‘animal model’; Kruuk and Hadfield 2007; Wilson et al. 2010), instead an appropriate approach is to compare matrices of genetic and song‐content similarity. Previous studies on the association between genetic similarity and song‐content similarity have shown mixed results with significant associations in some species (MacDougall‐Shackleton and MacDougall‐Shackleton 2001; Sosa‐López et al. 2013), but no association in others (Soha et al. 2004; Saranathan et al. 2007; González and Ornelas 2014). These heterogeneous findings suggest that additional factors, such as species‐specific learning differences (e.g., timing and extent of learning (Beecher and Brenowitz 2005)) or the specific genetic regions and vocal elements examined may play a role (Ribot et al. 2012; Ore et al. 2023; García et al. 2024; Marck et al. 2025).

As both genetics and song vary across spatial and temporal scales (Slater 1989; Hoffmann and Merilä 1999), a major source of variation in their relationship may arise from spatial and temporal heterogeneity of the data. For example, limited natal dispersal (Wheelwright and Mauck 1998; Ishibashi and Saitoh 2008; Szulkin and Sheldon 2008) can generate a relationship between genetic and geographic distance (Moore et al. 2005; Danner et al. 2017; Ore et al. 2023) that can vary among species (MacDougall‐Shackleton and MacDougall‐Shackleton 2001; McDonald 2009; Searfoss et al. 2020) or sexes (Graham et al. 2017; Cayuela et al. 2019). The spatial patterns of song similarity have been extensively studied (Marler and Tamura 1962; Koetz et al. 2007a), with dialects or clinal variation in song traits and degree of song sharing commonly reported (Janes and Ryker 2006; Budka et al. 2014; Lee et al. 2019) as a function of the geographical scale (Bradbury et al. 2001; Snijders et al. 2015; Graham et al. 2017). Typically, closer birds sing more similar songs (Beecher et al. 1994; Briefer et al. 2008; Price and Yuan 2011), although the opposite pattern has also been observed (Hultsch and Todt 1981; Laiolo 2011; Foote et al. 2013; Vargas‐Castro 2015). The drivers of geographic variation in song are not well established, but include sexual selection, signalling group membership, adaptation to habitat characteristics and cultural drift (Wright and Dahlin 2018). The timing and extent of song learning essentially influence the geographic pattern of song (Beecher et al. 1994; Nelson et al. 2001; Koetz et al. 2007b). Temporally, the genetic structure of the populations changes due to adaptive evolution or genetic drift (Kimura 1983), as does the song content due to genetic changes, but here cultural evolution and cultural drift can also accelerate modification (Garland and McGregor 2020; Otter et al. 2020; Souriau et al. 2025). The speed of temporal changes in song is probably influenced by song learning, song sharing patterns and vocalization complexity (Barta et al. 2023; Souriau et al. 2025). Therefore, because both song content and genetic similarity can vary spatially and temporally, controlling for geographic and temporal distance is crucial when investigating their associations.

Here, using the collared flycatcher ( *Ficedula albicollis* ) as a model species, a migratory passerine with complex and variable songs, we test whether song similarity reflects genome‐wide similarity within a population. Our aim was to ascertain whether song content provides information about genetic quality or compatibility for females, or instead primarily reflects culturally mediated learning. To address this, we compared matrices of song content and genome‐wide similarity using Mantel tests and phylogenetic methods, while controlling for geographic and temporal distances. Genome‐wide similarity was assessed by whole‐genome SNP data, providing higher resolution than most previous studies (but see Wilkins et al. 2018; Ore et al. 2023; Luo et al. 2024). Beyond the detailed genetic characterization, our study focused on a species with complex song and on the population scale that has been scarcely investigated, yet is crucial for understanding fine‐scale drivers and consequences of genetic variation.

## 2 Methods

### 2.1 Study Population and Song Recording Procedures

The collared flycatcher is a small (ca. 13 g), hole‐nesting and long‐distance migratory passerine that breeds in deciduous forests in Central Europe (Cramp and Perrins 1994). After spring migration (mid‐April), males establish a small territory around a nest hole (Cramp and Perrins 1994) and try to attract females by singing. Males typically sing intensively from soon after arrival until pairing (Pärt 1991; Garamszegi et al. 2004). Their repertoire is moderately high, containing 20–100 syllable types (estimated from 20 songs), and from these syllables variable songs are made up (Gelter 1987; Garamszegi et al. 2006; Zsebők, Herczeg, et al. 2018; see examples on Figure S12). Heritability of spectro‐temporal song traits and complexity is low in the species (Jablonszky, Canal, Hegyi, Herényi, et al. 2022), however, the genetic basis of song content has not been investigated. The collared flycatcher may be an open‐ended learner as repertoire size increases and the sequential organization of song changes with age (Garamszegi et al. 2007; Zsebők et al. 2021), and they may learn new syllables as adults (Vaskuti et al. 2016), although this was more clearly demonstrated in the sister species, the pied flycatcher ( *Ficedula hypoleuca* ) (Eriksen et al. 2011).

Data were collected between 2004 and 2018, as part of a long‐term study (Török and Tóth 1988), from a population of collared flycatchers breeding in the Pilis Mountains (47°43′N, 19°01′ E), Hungary. The study area is located in an unmanaged, oak‐dominated forest within the protected area of the Duna‐Ipoly National Park and contains ca. 800 nest boxes, mainly occupied by collared flycatchers. The maximum distance between nest boxes involved in this study is 1652 m.

Song recordings were made during the courtship period of the study species, between April 10 and May 10, following a standardized protocol (Garamszegi et al. 2006; Zsebők, Herczeg, et al. 2018). Briefly, each morning, during the peak singing activity (Pärt 1991; personal observations), we monitored the study area for newly arrived, unpaired birds. When a male was located holding a nest box and singing in its immediate vicinity, we recorded its song using a Telinga parabola dish with a Sennheiser ME62 microphone on a Tascam DR1, Microtrack II, or Zoom H4n handheld digital recorder (48 kHz sampling rate and 16‐bit quality). After the recordings, males were captured using a spring trap in their nest boxes. Birds were marked with individually numbered rings (official rings of the Hungarian Bird Ringing Centre). Blood samples were obtained from the brachial vein of the birds and stored in absolute ethanol. Additionally, blood samples were collected with the same method from birds captured during chick rearing in our nest box plots.

Recordings were conducted for at least 10 min and included a minimum of 20 songs (Zsebők et al. 2017). We have previously shown that the repertoire captured in 20 songs strongly correlates with the total song repertoire of males, that is, the number of different syllables they can produce (Garamszegi et al. 2002). Song recordings were conducted either after presenting a social stimulus (a live male or female decoy) for a short time to the focal male or without a stimulus. The different types of recording (with and without stimulus) were conducted randomly, and previous work showed that they affect the acoustic traits and song organization only marginally (Jablonszky, Canal, Hegyi, Krenhardt, et al. 2022; Zsebők et al. 2025). Thus, it is unlikely that the inclusion of both recording types caused systematic bias in further analyses. All recordings were performed in the absence of rain and strong wind, and songs with major disturbance from other birds, such as direct contact with conspecifics, were discarded from the analyses.

We manually selected the songs from the recordings using Adobe Audition 3.0 (Adobe Systems) software. Only songs with spectrograms clearly distinguishable from the background noise were considered. Syllables were manually selected, and from the syllable segments, we automatically extracted five spectrographic features using the Ficedula Toolbox (Zsebők, Blázi, et al. 2018): duration, minimum and maximum frequency, frequency bandwidth and mean frequency of the syllable. The latter variable was calculated as the mean of the peak frequency values in each spectrographic time window at the syllable level (Garamszegi et al. 2012). We grouped the syllables into 200 types (much larger than the individual repertoire size in the species) based on the five acoustic variables measured using the k‐means method in R (‘kmeans’ function in the ‘vegan’ R package; Oksanen et al. 2016; Zsebők, Blázi, et al. 2018).

In this study, we focus on song similarity based on the content of the song, that is, the syllable composition of the males' songs. The matrix of song similarity was built using a Bray‐Curtis‐based similarity measure (Bray and Curtis 1957), which accounts for both the number of common syllables of two males and the frequency of the syllables within the songs (Garamszegi et al. 2012). Specifically, similarity was calculated as $s=1-0.5\times \sum \left|o_{\mathrm{ik}}-o_{\mathrm{jk}}\right|$, where *o*_*ik*_ is the relative occurrence of syllable *k* (number of *k* syllables in the recording divided by the total number of syllables in the recording) in individual *i*, while *ojk* is that of the same syllable in individual *j*. With other similarity indices, such as the Jaccard index, songs with syllables ‘abc’ and ‘aaabc’ would be scored as fully similar, whereas our approach assigns a lower similarity value. We note that this approach does not capture variation in syllable order. However, sequence organization in the study species is known to be highly variable within individuals and context‐dependent (Zsebők et al. 2021, 2025). Thus, focusing on syllable composition allows us to capture a major axis of relevant variation related to syllable sharing and usage, while minimizing within‐individual noise in similarity estimates.

Overall, the song similarity matrix contained data from 122 males.

### 2.2 Genomic Data

DNA was extracted using a DNeasy Tissue Kit (Qiagen) and concentration was quantified using a Qubit Fluorometer (Life Technologies).

A paired‐end library (2 μg of genomic DNA per sample, digested with PstI) was prepared following the manufacturer's specifications at CNAG‐CRG (National Genome Analyses Centre) and sequenced on an Illumina HiSeq2000 v4 with 2 × 125 bp reads at a depth of approximately 10×. Library and sequencing conditions had been previously optimized in a pilot run, in which, among other aspects, 4 samples (8 μg DNA) were digested with several restriction enzymes (PstI, ApeKI). To evaluate the reliability of the sequencing process and optimize the assembly of loci, 20 individuals were included as duplicates.

Raw sequences were inspected with FASTQC (Andrews 2010) for quality control, demultiplexed and trimmed to remove the Illumina adapter and reads containing at least a single base with a Phred quality score of less than 10 (Toonen et al. 2013). We also removed sequences with a Phred quality score of less than 20 in more than 5% of the bases. A reference genome for the species (Ellegren et al. 2012) was used during assembly.

SNP calling was performed using bcftools (Danecek et al. 2016). We obtained 503.325 SNPs that were then filtered using PLINK 1.07 (Purcell et al. 2007). We removed loci that showed departures from Hardy–Weinberg equilibrium, were located on sex chromosomes, had minor allele frequency below 0.05 or a maximum per‐SNP missing of 10%. Individuals with more than 5% of missing data were excluded. The final dataset contained 188.231 biallelic SNPs. The sample size for these analyses was 726 males for which we could obtain genetic data, and these included 100 with information on song.

We assessed genome‐wide similarity between individuals using two approaches. First, we built a matrix of pairwise relatedness based on IBS (Identity By State) segments in PLINK 1.07. Second, we performed Classical Multidimensional Scaling (MDS) on the IBS matrix and clustered the individuals using a Gaussian mixture model. The best fitting model was selected using Bayesian information criterion (BIC) with the number of clusters estimated by the function ‘Mclust’ (library ‘mclust’; (Scrucca et al. 2023)), in the R software, v.4.1.3. Both IBS distances and genetic clusters capture genetic differences among individuals, but they do so in different ways. IBS measures direct genomic similarity between pairs of individuals without accounting for underlying population structure or distinguishing similarity due to ancestry from that arising by chance. In contrast, clustering approaches can capture hidden population structure, reflect evolutionary history and gene flow and, by grouping individuals according to shared genetic background, reduce noise and increase statistical power (Rosenberg et al. 2001; Li et al. 2011). In an initial step, clustering was performed on the full genetic dataset, yielding 13 clusters with good assignment reliability (mean assignment uncertainty = 0.052 ± 0.101, range: 0–0.538; maximum overlap = 0.018). However, these clusters did not accurately characterize the subset of individuals for which song data was available as within‐cluster genetic distances were slightly greater than between‐cluster distances (mean: 0.252 ± 0.005 and mean: 0.251 ± 0.004, respectively). Thus, to improve classification accuracy, we repeated the genetic clustering using only the birds with song data, which yielded 4 well‐defined clusters with high reliability (mean uncertainty: 0.017 ± 0.044, range: 0–0.245; maximum overlap = 0.011). By restricting clustering to individuals for which song recordings were available, we ensured internally consistent genetic grouping with adequate sample sizes for meaningful genotype–phenotype comparisons.

### 2.3 Statistical Analyses

Statistical analyses were performed in R v.4.1.3 (R Core Team 2023).

To test the relationship between song content and genome‐wide similarity we used the song similarity and IBS matrices described above. To account for spatial and temporal effects, we constructed geographic and year distance matrices. Geographic distance was based on nest‐box coordinates at the time of song recording (mean: 637.400 ± 420.795, range: 0–1651.5 m), as these locations best represent the socio‐ecological context in which songs were produced and are therefore more relevant for song expression than alternative coordinates for the same individuals (e.g., at natal or first breeding nest‐boxes; see Text S1 for details). The temporal distance matrix was calculated as the difference in years between recordings of each pair of birds (mean: 5.111 ± 3.849, range: 0–13). The geographic and year distance matrices were log‐transformed and scaled to values between 0 and 1 to ensure biological interpretability (Houle et al. 2011) and comparability with the other matrices. All the matrices (genetic, song, geographic and year) were transformed to distances before analyses, although for simplicity we refer to (song or genome‐wide) ‘similarity’ throughout the text. As the dataset was skewed (contained few related individuals), genetic distance values lower than 0.23 were considered strongly related individuals and were discarded from all analyses (0.4% of the data; see Figure S1). We conducted all analyses on the subset of birds possessing both genetic and song data (*N* = 100).

Before analysing whether song similarity between individuals was related to their genetic similarity, the main aim of this study, we conducted a set of preliminary analyses to avoid misleading conclusions, for example, due to temporal or spatial genetic structure. First, we used linear mixed models (LMM) to examine whether genetic distance differed between immigrants (i.e., first‐time banded as adults) and recruits (i.e., banded as fledglings), including pairwise genetic distance as the response variable, the joint immigrant status of the ‘pair’ as a fixed factor (recruit‐recruit, recruit‐immigrant or immigrant‐immigrant) and individual identity of the two birds as random intercepts. Regarding genetic clusters, we used permutation test to assess whether they varied according to immigration status. Second, we investigated potential temporal effects, testing (i) whether genetic distance between pairs of individuals increased with the time interval between the sampling years or due to a linear trend over the study period. To this end, we fitted a LMM with pairwise genetic distance as the response variable, including the interval between the sampling years (z‐ and log‐transformed) and the median year of the measurements (z‐transformed) as fixed factors and individual identities as random intercepts. (ii) We repeated this model with song distance as the response variable to assess temporal change in song content. (iii–iv) In addition, we used Mantel tests (R package ‘vegan’ (Oksanen et al. 2020)) to check the association between the year distance matrix with both the song and IBS matrices. (v) In the latter case, we also performed permutation test to assess whether the distribution of genetic clusters varied among years. Third, we investigated spatial effects using Mantel tests to examine whether geographical distances were related to (i) song similarity or (ii) genetic distance (IBS matrix). (iii) Additionally, to confirm that geographic patterns were not underlying the potential relationship between genetic cluster and song, we tested whether geographic distance predicted cluster assignments using phylogenetic methods (see below).

To evaluate whether song similarity between individuals was related to their genome‐wide similarity, the main aim of this study, we used both the IBS matrix and genetic clusters. When working with matrices, we run two types of tests: (i) a simple Mantel test to directly test the relationship between the IBS matrix and the song similarity matrix and (ii) two partial Mantel tests to test such relationship while controlling for either geographic or year distances. When working with genetic clusters, we used a phylogenetic approach to test whether song similarity was higher within than between genetic clusters. Under this phylogenetic approach, the relatedness between the individuals can be modelled as a phylogenetic tree derived from their song similarity (see similar methodology in: Ranjard et al. 2015; Arató and Fitch 2021) and the genetic clusters are treated as a discrete trait mapped onto the tree, allowing us to test whether cluster membership shows a phylogenetic signal. We used Pagel's lambda (*λ*) and the fitDiscrete function in the R package ‘Geiger’ to test for a phylogenetic signal (Harmon et al. 2008), that is whether birds with similar song contents tend to belong to the same genetic cluster. Pagel's *λ* ranges from 0 to 1; a value of 0 indicates that no phylogenetic signal exists, whereas a value of 1 indicates that the focal trait gradually accumulates changes over time according to a Brownian motion process. Two phylogenetic models were fitted using the ARD (‘all‐rates‐different’) transition model: one with the original tree and one with a tree where *λ* was constrained to be 0 using the ‘rescale’ function in ‘phytools’ (Revell 2012). Then, a likelihood ratio test (LRT) was used to assess whether *λ* differed from zero (Pagel 1999). If significant, as the function did not directly estimate *λ*, we compared models with constrained *λ* values between 0 and 1 (with 0.1 increments) and selected the value from the model with the highest likelihood as the approximate *λ*. Furthermore, we employed an alternative statistical test to investigate directly whether the song distances between the birds belonging to the same genetic cluster was smaller than song distances between birds belonging to different clusters. For this analysis, we used linear mixed models (LMM), including the song distance between pairs of individuals as response variable, whether the two birds belonged to the same or different clusters as fixed factor and individual identity of the two birds as random intercepts.

### 2.4 Ethical Note

All applicable international, national and/or institutional guidelines for the care and use of animals were followed. The study was approved by the ethical committee of the Eötvös Loránd University (no TTK/2203/3). Housing conditions of decoys, handling and behavioural assays were conducted in a way to minimize the welfare impact on the birds. The time required for these procedures was restricted to a minimum and all of them were carried out as cautiously and efficiently as possible. Permissions for the fieldwork were provided by the Middle‐Danube Valley Inspectorate for Environmental Protection, Nature Conservation and Water Management and the National Scientifc Ethical Committee on Animal Experimentation and the Department of Environment and Nature Protection of the Hungarian Government Office (numbers: KTVF 16360–2/2007, KTVF 30871–1/2008, KTVF 43355–1/2008, KTVF 45116–2/2011, KTVF 21664–3/2011, KTVF 12677–4/2012, KTVF 10949–8/2013, KTF 11978–5/2015, PEI/001/1053–6/2015, PE/EA/101–8/2018, PE‐06/KTF/8550–4/2018 and PE‐06/KTF/8550–5/2018).

## 3 Results

### 3.1 Assessing Spatial and Temporal Structure in Genetics and Song Content

Regarding immigrant‐recruit status, pairwise genetic distance between recruits was slightly lower than distances between immigrants (estimate −0.002, confidence interval (CI): (−0.003, −0.0003)), although the LRT test for the overall effect of the factor was not significant (*χ* ^2^ = 3.744, *p* = 0.154; see details and figure in the Table S1, Figure S2). Similarly, permutation tests indicated no significant difference in the frequencies of genetic clusters between immigrants and recruits, regardless of whether clusters were based on the whole dataset (*χ* ^2^ = 16.594, *p* = 0.166, Cramér's V = 0.407, Figures S3 and S4) or the restricted dataset (*χ* ^2^ = 1.314, *p* = 0.7495, Cramér's V = 0.116, Figure S5).

Concerning temporal effects, we found that both the genome‐wide and song distances between pairs of individuals increased with the time interval between their sampling years (genetic distance: 0.0002, CI: (0.0001, 0.0002), *χ* ^2^ = 52.711, *p* < 0.001; song distance: 0.002, CI: (0.002, 0.003), *χ* ^2^ = 21.119, *p* < 0.001). Genetic distance also increased with the median of the two measurement years (0.002, CI: (0.002, 0.002), *χ* ^2^ = 43.338, *p* < 0.001; see Table S2, Figure S6). Regarding the year distance matrix, we did not find a relationship with genetic distance (*N* = 100, Mantel *r* = −0.012, *p* = 0.718), but found a positive correlation with song distance (Mantel *r* = 0.059, *p* = 0.001, Figure S7). The permutation test showed no significant variation in genetic cluster frequencies among years, regardless of whether based on the full dataset (*χ* ^2^ = 212.340, *p* = 0.107, Cramér's V = 0.425, Figures S8 and S9) or the restricted dataset (*χ* ^2^ = 45.926, *p* = 0.333, Cramér's V = 0.395, Figure S10). Overall, these results indicated that time between the measurements did not clearly affect genome‐wide similarity, whereas song similarity consistently declined with increasing time between recordings.

Regarding geographic effects, there was no correlation between the genetic distance of individuals and the geographic distances of their nest boxes in the year of the song recording (*N* = 99, Mantel *r* = 0.021, *p* = 0.197). Geographic distance was not associated with the probability of belonging to different clusters, that is *λ* did not differ from 0 for clusters based on either the full dataset (*χ* ^2^ = −217.301, *p* = 1) or the restricted dataset (*χ* ^2^ = −194.176, *p* = 1). Likewise, song content similarity was not correlated with the geographic distance of the nest boxes at the time of song recording (Mantel *r* = −0.004, *p* = 0.585).

### 3.2 The Relationship Between Song Similarity and Genome‐Wide Similarity

**FIGURE 1:**
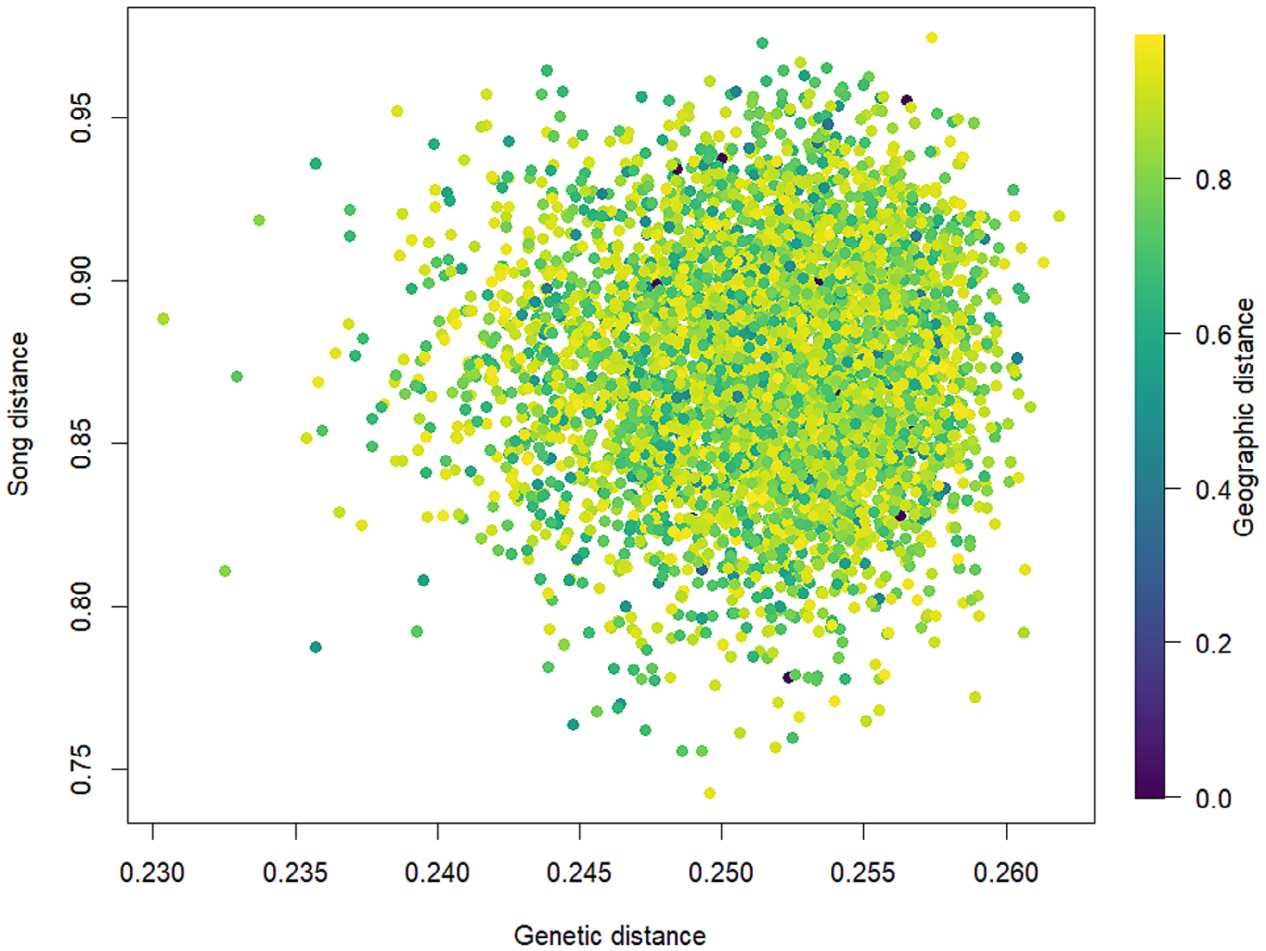
No significant relationship was detected between genetic distance, song distance and geographic distance. Geographic distance is depicted as colours.

**FIGURE 2:**
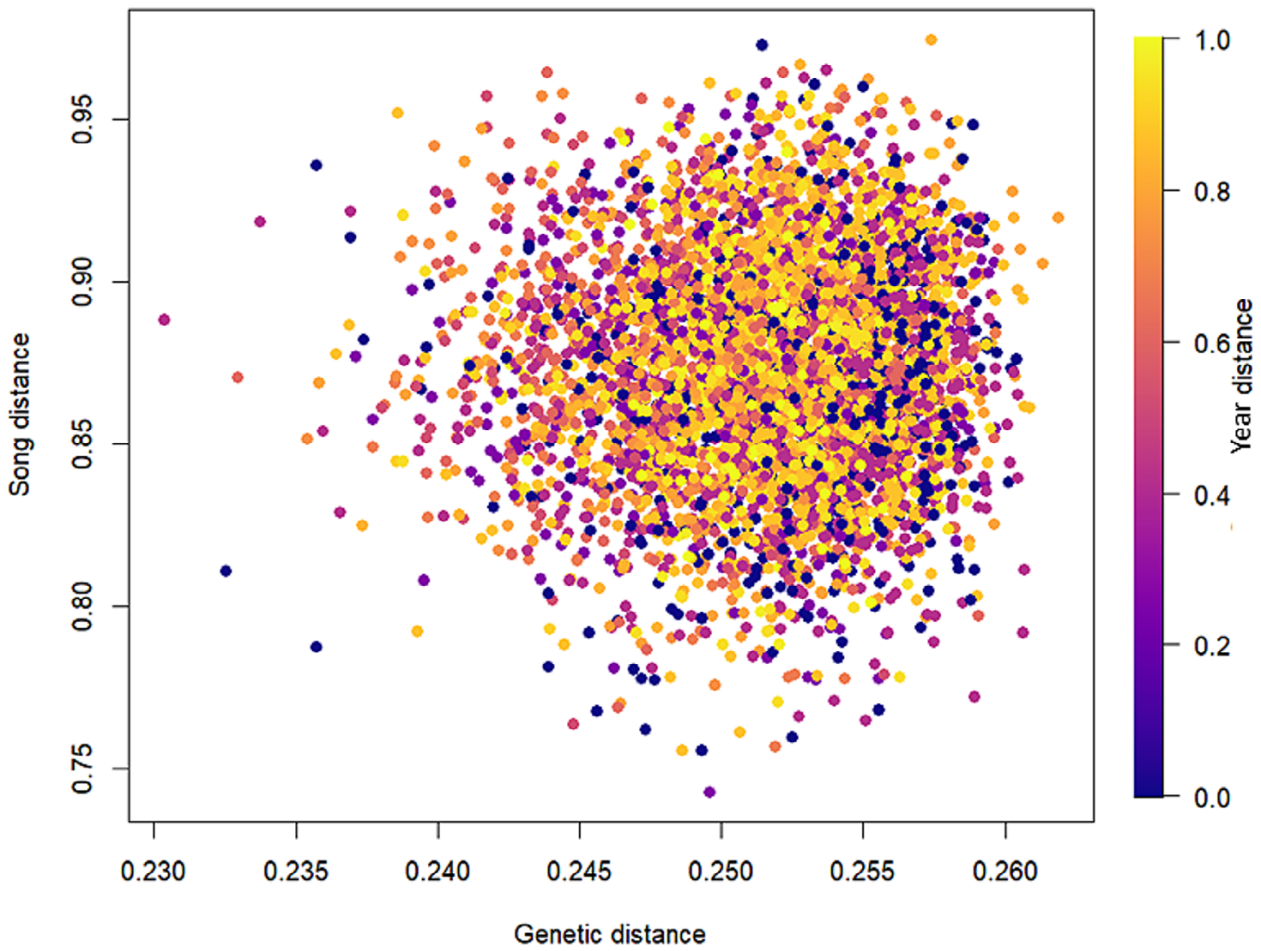
No significant relationship was detected between genetic and song distance, but a significant association was found between song distance and years elapsed between the recordings (year distance), the latter depicted as colours.

**FIGURE 3:**
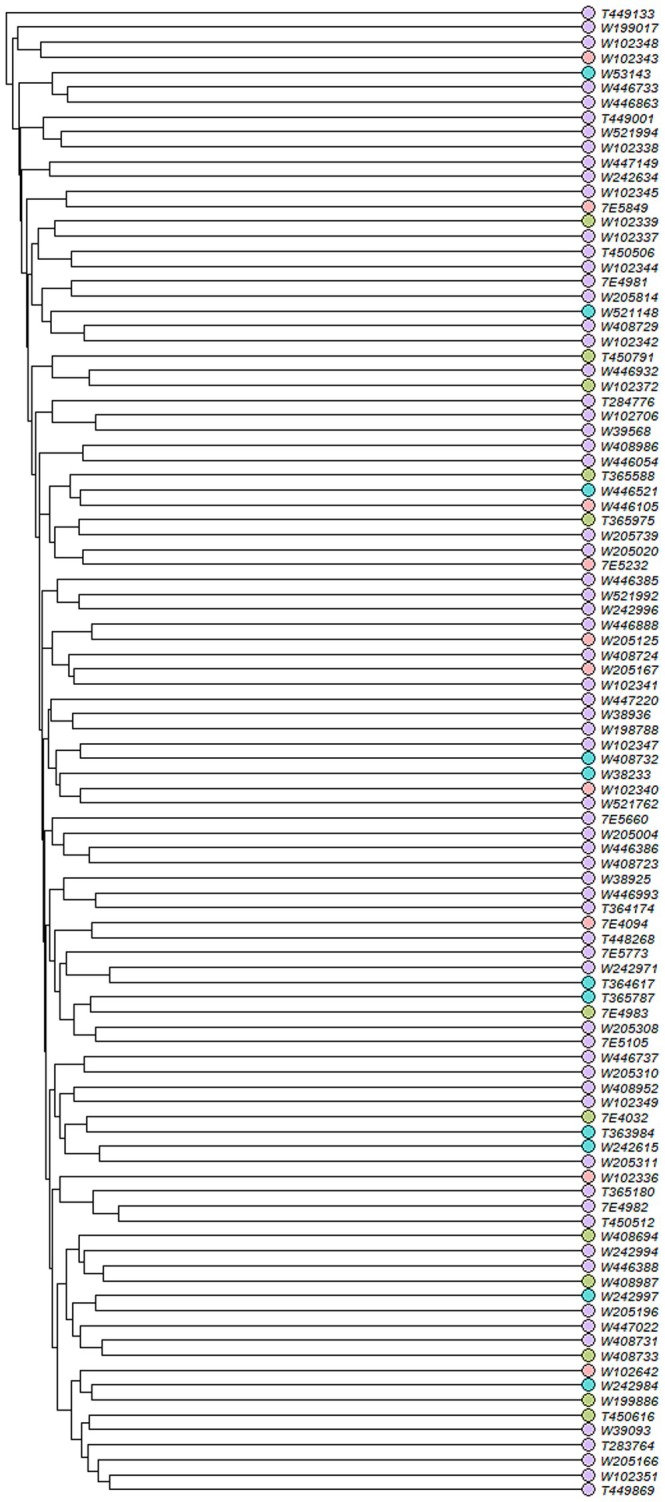
Lack of relationship between song distance and genetic clusters based on the restricted dataset with song data. The colours depict the 4 genetic clusters.

Neither the simple Mantel test between the IBS and song matrices (Mantel *r* = 0.014, *p* = 0.409, Figure S11), nor the partial Mantel tests controlling for the effect of geographic distance (*N* = 100, Mantel *r* = 0.013, *p* = 0.431, Figure 1) or temporal distance (Mantel *r* = 0.015, *p* = 0.405, Figure 2) were significant. When genetic clusters based on the full dataset were used, individuals belonging to the same genetic cluster had more similar songs than those belonging to different genetic clusters (*χ* ^2^ = 212.340, *p* < 0.001, *λ*≈1). However, the relationship disappeared when clusters were based on the restricted dataset, where *λ* could not be differentiated from 0 (*χ* ^2^ = 0.983, *p* = 0.322; Figure 3). In the LMM investigating song distances between birds belonging to the same or to different clusters, within‐ and between‐cluster song distances did not differ either in the case of clusters based on the whole dataset (estimate: −0.001, confidence interval (CI): (−0.003, 0.001), LRT test testing the effect of the whole factor: *χ* ^2^ = 1.832, *p* = 0.176), or on the restricted dataset (estimate: 0.0003, CI: (−0.002, 0.003), LRT test: *χ* ^2^ = 0.082, *p* = 0.774). Overall, these analyses provide no consistent support for an association between genome‐wide similarity and song similarity.

## 4 Discussion

Using 15 years of song recordings together with a large panel of genome‐wide SNPs from a wild migratory species with complex song, we found no robust evidence that male song similarity reflects genome‐wide similarity. Genome‐wide similarity was unrelated to either geographic or temporal distance, indicating a lack of detectable spatial or temporal genetic structure within the study population. Likewise, geographic distance did not predict song similarity, suggesting that males do not primarily learn songs from or match songs to neighbours. Instead, song content similarity decreased with the time elapsed between the recordings, suggesting that song variation accumulates through time independently of genome‐wide genetic divergence and likely reflects processes acting on culturally transmitted traits. Together, these findings show that genetic influences on biologically relevant song features are minimal.

The loss of significance of the relationship between song and genome‐wide similarity when clustering was restricted to individuals with song data suggests that the initial association was driven by large‐scale population structure rather than by genetic differentiation relevant to song variation at the individual level. Overall, the lack of consistent association across analyses does not support the idea that song composition conveys genetic information that could be used in sexual and social contexts. Instead, our findings align with extensive research showing that male song in oscine birds is extremely flexible and strongly shaped by social influences throughout ontogeny (Price 1979; Mets and Brainard 2018; Jablonszky, Canal, Hegyi, Herényi, et al. 2022). Particularly, in collared flycatchers song expression is strongly modulated by song learning and other social and environmental factors (Jablonszky, Canal, Hegyi, Herényi, et al. 2022; Jablonszky, Canal, Hegyi, Krenhardt, et al. 2022; Krenhardt et al. 2025; Zsebők et al. 2025). Vocal learning enables males to rapidly adjust their songs to the local social environment (Beecher and Brenowitz 2005) and to the transmission conditions of the habitat (Morton 1975). In general, learning from neighbours, and consequently sharing similar songs within local groups or neighbourhoods may confer various advantages during territory defence (Wilson and Vehrencamp 2001; Briefer et al. 2008; Draganoiu et al. 2014) and during mate choice (Soma 2011; Baker et al. 1987; Mortega et al. 2014; Slabbekoorn and Smith 2002), but in particular in our study species learning from neighbours is not likely (Garamszegi et al. 2012; Jablonszky et al. 2025). These processes illustrate how social learning and the cultural transmission can drive spatial and temporal variation in song content independently of genetic differentiation with potential long‐term consequences for population divergence and speciation (Slabbekoorn and Smith 2002; Lachlan and Servedio 2004).

Previous work exploring the association between genetic relatedness and song similarity have yielded mixed results, both for spectro‐temporal traits (Irwin et al. 2008; Acero‐Murcia et al. 2021; Ore et al. 2023) and for song content (Soha et al. 2004; Sosa‐López et al. 2016). However, only few studies have measured this relationship using SNPs (Lipshutz et al. 2017; Wilkins et al. 2018) and, to our knowledge only one, aside from our own, has relied on a large number of genome‐wide SNPs (> 100.000). That study, conducted on the wood warbler *Setophaga townsendi* , a migratory species with simple song, found only a weak song‐genetic relationship, consistent with our findings (*N* = 71; Ore et al. 2023). Apart from methodological differences, discrepancies among studies can arise from variation in the spatial scales considered relative to the scale of dialect or song variation, which in turn depends on the dispersal propensity of the species (Kroodsma et al. 1999; Bradbury et al. 2001; Marck et al. 2025). Most of the above studies have focused on large spatial scales and inter‐population comparisons, whereas fine‐scale, within‐population studies are comparatively scarce. These fine‐scale studies found no relationship between genetic similarity and the mimicked‐species repertoires in Icterine warblers ( *Hippolais icterina* ; Riegert and Juzlová 2018), or the dialects of orange‐tufted sunbirds ( *Nectarinia osea* ) within a 1.5 km^2^ area (Leader et al. 2008). Together, these studies, in line with our findings, suggest that song and genetic similarity are (often) decoupled at the population scale. Temporal effects may further contribute to discrepancies across studies, as songs recorded over long periods may gradually diverge through cultural or genetic processes (Luther and Baptista 2010; Garland and McGregor 2020; Otter et al. 2020). In our case, the association between song and genome‐wide similarity was unlikely to be confounded by either geographic or temporal distance between the recordings, because genetic clusters did not vary among years and were unrelated to geographical distance. Additionally, partial Mantel tests controlling for geographical or temporal effects yielded results consistent with pairwise comparisons. Although our sample size (100 individuals) may have limited statistical power to detect subtle associations with complex tests, it is comparable to that of studies reporting song‐genetic associations (*N* = 54–215; MacDougall‐Shackleton and MacDougall‐Shackleton 2001; Irwin et al. 2008; Sosa‐López et al. 2013), though direct comparison is difficult because most studies compared sampling sites rather than individuals.

Temporal and spatial factors can both shape variation in bird song and genetic structure (Slater 1989; Hoffmann and Merilä 1999), yet in our population their influence was limited and asymmetric. Temporal effects influenced song but not genome‐wide variation, as genetic distance showed no clear changes over time, while song similarity declined with increasing temporal separation between recordings. In species with learned vocalizations, such temporal accumulation of song differences is expected when songs are transmitted culturally across generations (Ince et al. 1980; Williams et al. 2013; Ju et al. 2019). Because song learning plays a major role in oscine birds (Beecher and Brenowitz 2005) and song heritability appears limited in our study species (Jablonszky, Canal, Hegyi, Herényi, et al. 2022), the observed pattern suggests that song variation accumulates largely independently of genome‐wide genetic divergence. The observed temporal divergence does not identify the specific mechanisms responsible for song change. Cultural drift, innovation and other processes associated with cultural evolution may all contribute to the gradual modification of song traditions through time (Otter et al. 2020; Martin et al. 2023; Souriau et al. 2025). In addition, selective forces acting on song, including sexual selection (Candolin and Heuschele 2008; Snell‐Rood and Badyaev 2008; Xing et al. 2017), adaptation to changing acoustic environments (Morton 1975; Goretskaia et al. 2018) and morphological changes affecting song production such as in body or beak size (Badyaev et al. 2008; Mikula et al. 2021; Sagar et al. 2024) may influence which song variants are retained and spread. Thus, while the temporal pattern is consistent with cultural evolution, the present data do not allow us to distinguish among the specific cultural and selective mechanisms generating song change through time. Regarding geographic factors, often associated with song variation (González and Ornelas 2014; Riegert and Juzlová 2018; Ore et al. 2023) and less frequently with genetic structure in other species (Moore et al. 2005; Nicholls et al. 2006; Ore et al. 2023), they did not predict either genome‐wide similarity or song similarity in this population. The absence of spatial genetic structure suggests that individuals settle randomly with respect to relatedness. Likewise, the lack of geographic patterns in song indicates that males do not primarily learn or incorporate song elements from nearby conspecifics (Beecher et al. 1994; Liu and Kroodsma 2006; Price and Yuan 2011), unlike that reported at comparable spatial scales in other species (Koetz et al. 2007b; Akcay et al. 2014; Barišić et al. 2018), and implies that geographical isolation mediated by song is unlikely in the study population (MacDougall‐Shackleton and MacDougall‐Shackleton 2001; Shieh 2004; Janes and Ryker 2006). Contrary to our current result, previous work in collared flycatchers found that close males differ in specific song traits such as minimum frequency and repertoire size (Jablonszky et al. 2025), as well as that song similarity increases with distance at a small spatial range of a few hundred meters (Garamszegi et al. 2012). Collectively, these findings suggest that males may adjust certain song traits to differentiate from their neighbours (Laiolo 2011; Garamszegi et al. 2012; Torti et al. 2017), while overall song content remained unaffected at higher spatial scales (roughly 2 km^2^). Species‐specific life‐history traits, including long‐distance migration (Kroodsma et al. 1999; Nelson et al. 2001; Yoon et al. 2013), short lifespan and substantial immigration (Jablonszky et al. 2025), likely reduce the advantages of local dialects (Beecher et al. 1994; Leader et al. 2008; Nelson and Poesel 2009) in our study species, while methodological differences among studies may further contribute to inconsistencies in spatial patterns (Ribot et al. 2012). Overall, our results indicate that spatial factors contribute minimally to variation in either genome‐wide similarity or song content at the investigated spatial scale.

In conclusion, we found no association between song content similarity and genome‐wide genetic similarity within a population of a passerine species with complex song. These findings suggest that, at the spatial and temporal scale examined, song content does not provide a reliable proxy for overall genomic relatedness. Our findings contribute to a growing body of work showing that cultural processes and social learning dominate population‐level patterns of song variation in oscine birds. Importantly, our study highlights species‐ and scale‐dependent differences in how genetic variation relates to song and underscores the value of genome‐wide data for accurately assessing genetic contributions to behavioural variation. Future research integrating fine‐scale genomic analyses targeting functionally relevant loci, together with longitudinal and experimental approaches, will be essential to disentangle how genetic and non‐genetic factors jointly contribute to song variation.

## Author Contributions

L.Z.G. and D.C. designed the study, all authors except D.C. participated in the field work, M.J., L.Z.G., G.N. and S.Z. collected and analysed song recordings, D.C. performed genetic analyses and obtained the genetic relatedness matrix of individuals, D.C. and J.M. carried out the statistical analysis with inputs from L.Z.G. and wrote the first draft of the manuscript. All authors contributed to the revisions of the manuscript.

## Funding

This work was supported by funds from the Hungarian National Research, Development and Innovation Office (K139992, FK146466). DC was supported by a Talent Attraction fellowship from the Autonomous Community of Madrid (CAM), Spain (2022‐T1_AMB‐24025) and the project PID2022‐141763NA‐I00 and CNS2024‐154721, funded by MCIN/AEI (https://doi.org/10.13039/501100011033).

## Disclosure

Benefit‐Sharing: Benefits Generated: Benefits from this research accrue from the sharing of our data and results on public databases as described above. More broadly, our group is committed to international scientific partnerships, as well as institutional capacity building.

## Conflicts of Interest

The authors declare no conflicts of interest.

## Supporting information


**Text S1:** We considered originally three geographical distance measures based on the nest box coordinates of individuals (i) at birth, (ii) at the first breeding attempt and (iii) where the song recording was made. However, because we had limited data for nest box at birth overlapping with song data, and as the Mantel correlation of these three matrices were significant (albeit small in two cases; birth‐first breeding: Mantel *r* = 0.036, *p* = 0.036, *N* = 290; birth‐recording: Mantel *r* = 0.071, *p* = 0.009, *N* = 68; first breeding‐recording Mantel *r* = 0.676, *p* = 0.001, *N* = 110), we used only distance at the year of recording (most relevant for song) in the further analyses.
**Figure S1:** Distribution of the genetic distance of the population. Based on this histogram values below 0.23 (values between related individuals) were discarded from the analyses.
**Table S1:** Results of the linear mixed model investigating the effect of immigrant or recruit status on genetic distances. Estimates for fixed effects and variance for the random effects with 95% confidence intervals are displayed. Fixed effects of which the confidence interval does not include zero are in bold. Number of observations = 4746, number of birds = 97.
**Figure S2:** Genetic distances according to the immigrant or recruit status of the two birds.
**Figure S3:** Permutation test checking the distribution of genetic clusters based on the whole dataset between immigrant and recruit birds.
**Figure S4:** Genetic clusters based on the whole dataset of immigrant and recruit birds.
**Figure S5:** Permutation test checking the distribution of genetic clusters based on the restricted dataset between the years.
**Table S2:** Results of linear mixed models investigating the effect of year difference and median of the two measurement years on genetic and song distances. Estimates for fixed effects and variance for the random effects with 95% confidence intervals are displayed. Fixed effects of which confidence intervals do not include zero are in bold. Genetic distance: number of observations = 4746, number of birds = 97; song distance: number of observations = 7381, number of birds = 121.
**Figure S6:** The relationship between genetic distance and the median of the two measurement years.
**Figure S7:** Relationship between genetic or song distance and year distance.
**Figure S8:** Permutation test checking the distribution of genetic clusters based on the whole dataset between the years.
**Figure S9:** The distribution of genetic clusters based on the whole dataset among years.
**Figure S10:** Permutation test checking the distribution of genetic clusters based on the restricted dataset between the years.
**Figure S11:** Relationship between song distance and genetic distance.
**Figure S12:** Example songs from different individuals.

## Data Availability

Raw sequence reads are deposited in the SRA (BioProject PRJNA1067851). Data and code used for the statistical analyses and corresponding metadata is deposited in Figshare (10.6084/m9.figshare.31354018).
