# Supplementary material for: Genetic Composition Is Unrelated to Song Content in a Wild Passerine
Source: Mol Ecol. 2026 Aug 5;35(15):e70491. doi: 10.1111/mec.70491 (PMC13439345; doi:10.1111/mec.70491)
Supplement: Supplementary file 1 [file MEC-35-e70491-s001.docx]

Supplementary material for: **Genetic composition is unrelated to song content in a wild passerine**

by Mónika Jablonszky, David Canal, Gergely Hegyi, Gergely Nagy, Balázs Rosivall, János Török, Sándor Zsebők, László Zsolt Garamszegi

Text S1: We considered originally three geographical distance measures based on the nest box coordinates of individuals i) at birth, ii) at the first breeding attempt, and iii) where the song recording was made. However, because we had limited data for nest box at birth overlapping with song data, and as the Mantel correlation of these three matrices were significant (albeit small in two cases; birth-first breeding: Mantel r = 0.036, p = 0.036, N = 290; birth-recording: Mantel r = 0.071, p = 0.009, N = 68; first breeding-recording Mantel r = 0.676, p = 0.001, N = 110), we used only distance at the year of recording (most relevant for song) in the further analyses.

Fig S1: Distribution of the genetic distance of the population. Based on this histogram values below 0.23 (values between related individuals) were discarded from the analyses

Table S1: Results of the linear mixed model investigating the effect of immigrant or recruit status on genetic distances. Estimates for fixed effects and variance for the random effects with 95% confidence intervals are displayed. Fixed effects of which the confidence interval does not include zero are in bold. Number of observations = 4746, number of birds = 97

|  | Estimate (CI) |
| --- | --- |
| Fixed effects |  |
| Recruit-immigrant | -0.001 (-0.002, 0.0003) |
| Immigrant-immigrant | **-0.002** (-0.003, -0.0003) |
| Random effects |  |
| ID of bird1 | <0.001 (<0.001, <0.001) |
| ID of bird2 | <0.001 (<0.001, <0.001) |
| Residual | <0.001 (<0.001, <0.001) |

Fig S2: Genetic distances according to the immigrant or recruit status of the two birds

Fig S3: Permutation test checking the distribution of genetic clusters based on the whole dataset between immigrant and recruit birds

Fig S4: Genetic clusters based on the whole dataset of immigrant and recruit birds

Fig S5: Permutation test checking the distribution of genetic clusters based on the restricted dataset between the years

Table S2: Results of linear mixed models investigating the effect of year difference and median of the two measurement years on genetic and song distances. Estimates for fixed effects and variance for the random effects with 95% confidence intervals are displayed. Fixed effects of which confidence intervals do not include zero are in bold. Genetic distance: number of observations = 4746, number of birds = 97; song distance: number of observations = 7381, number of birds = 121

|  | Genetic distance | Song distance |
| --- | --- | --- |
|  | Estimate (CI) | Estimate (CI) |
| Fixed effects |  |  |
| Year difference | **0.0002** (0.0001, 0.0002) | **0.002** (0.002, 0.003) |
| Median year | **0.002** (0.002, 0.002) | -0.001 (-0.004, 0.001) |
| Random effects |  |  |
| ID of bird1 | <0.001 (<0.001, <0.001) | 0.0002 (0.0002, 0.0003) |
| ID of bird2 | <0.001 (<0.001, <0.001) | 0.0002 (0.0002, 0.0002) |
| Residual | <0.001 (<0.001, <0.001) | 0.0007 (0.0007, 0.0007) |

Fig S6: The relationship between genetic distance and the median of the two measurement years

Fig S7: Relationship between genetic or song distance and year distance

Fig S8: Permutation test checking the distribution of genetic clusters based on the whole dataset between the years

Fig S9: The distribution of genetic clusters based on the whole dataset among years

Fig S10: Permutation test checking the distribution of genetic clusters based on the restricted dataset between the years

Fig S11: Relationship between song distance and genetic distance

Fig S12: Example songs from different individuals
